# Spindle Cell Hemangioma of the Lung: A Case Report

**DOI:** 10.7759/cureus.21191

**Published:** 2022-01-13

**Authors:** Abhishek Nimkar, Michael Mandel, Arzu Buyuk, Christos Stavropoulos, Ashutossh Naaraayan

**Affiliations:** 1 Internal Medicine, Montefiore New Rochelle Hospital, Albert Einstein College of Medicine, New Rochelle, USA; 2 Internal Medicine/Pulmonary and Critical care, Montefiore New Rochelle Hospital, Albert Einstein College of Medicine, New Rochelle, USA; 3 Pathology, Northern Westchester and Phelps Hospital, Hofstra Northwell School of Medicine, Westchester, USA; 4 Thoracic Surgery, Montefiore New Rochelle Hospital, Albert Einstein College of Medicine, New York City, USA

**Keywords:** vascular tumor, benign tumor, lung tumor, rare presentation, spindle cell hemangioma

## Abstract

Spindle cell hemangioma (SCH) is an uncommon tumor that usually presents as a subcutaneous or deep dermal nodule affecting the extremities of young people. It is primarily a benign vascular neoplasm with a tendency to recur locally. Reports describing SCH diagnosed in muscles, retroperitoneum, mediastinum, and even in the spinal cord occasionally surface in the literature. We report a very rare case of SCH diagnosed in the lung.

## Introduction

Spindle cell hemangioma (SCH) is a rare benign tumor characterized by cavernous blood vessels and spindled areas that typically arise in the subcutis of the distal extremities, particularly the hand, or under the mucous membranes. These tumors are more common in a younger population, and, although they have low malignant potential, they tend to recur locally. Previously only one case of SCH in the lungs has been reported [1]. Herein, we present the case of a 62-year-old woman with gradually progressing multiple pulmonary nodules, suspicious for adenocarcinoma, who was diagnosed with SCH.

## Case presentation

**Figure 1:**
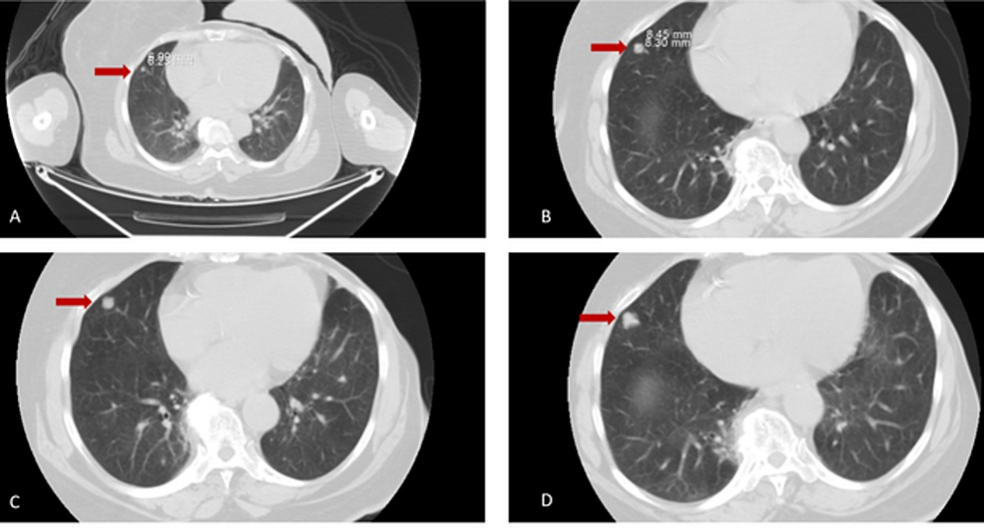
Timeline of progression of the nodule from 2013 (A), 2014 (B), 2016 (C), and 2018 (D).

This 62-year-old female had a history of left breast cancer for which she underwent a mastectomy in 2006, hypercholesterolemia, hypertension, and a smoking history of one pack per week from her teens to her mid-50s (approximately six pack-years). She was incidentally found to have a 7-mm pulmonary nodule in her right middle lobe and multiple smaller nodules bilaterally during surveillance computed tomography (CT) scan in 2009. She had a chronic dry cough and dyspnea on exertion but denied any other symptoms including chest tightness, fever, or weight loss. Pulmonary function tests showed preserved lung function. In 2013, she was diagnosed with and underwent surgery for granulosa theca cell tumor of the neck. During the workup, a positron emission tomography (PET) scan was done, which measured the largest pulmonary nodule in the right middle lobe at 9 mm and multiple small nodules. This nodule did not take up fluorodeoxyglucose and thus was considered metabolically inactive. The nodule had smooth borders with uniform density and did not have any features suggestive of malignancy on imaging. Based on patient preference, a decision was made to follow up the nodules with subsequent CT scans. Between 2013 and 2018, the nodule gradually increased to 13 mm in size (Figures 1A-1D). The morphology of the nodule changed to an irregular pattern which became suspicious for malignancy. After discussing with the patient, a video-assisted thoracoscopy and right middle lung lobectomy were performed.

**Figure 2:**
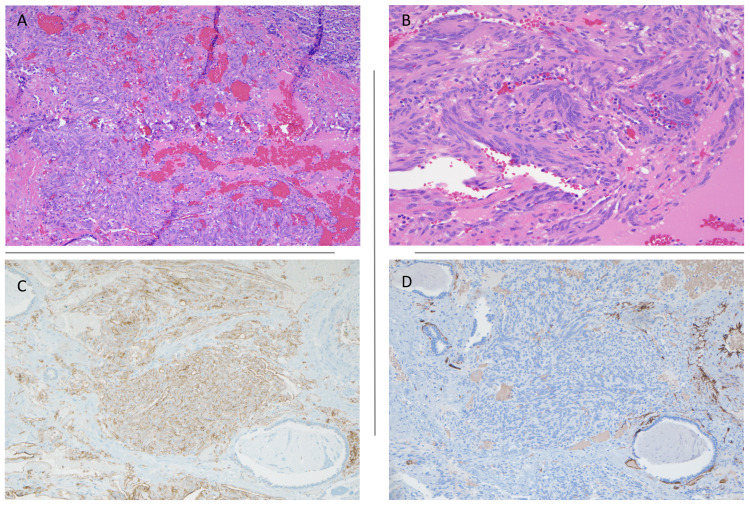
Tumor showing dilated blood vessels, H&E x4 (A) and bland spindle cell proliferation, H&E x20 (B); immunohistochemical staining with CD31 (C) and negative staining with CD34 (D).

Gross examination showed a red-tan soft oval tumor with cystic areas. Microscopically the tumor was composed of bland spindle cell proliferation, dilated blood vessels, hemosiderin-laden macrophages, and calcifications (Figure 2A). The spindle cells had scant cytoplasm, uniform nuclei without mitosis or necrosis, and were arranged in small clusters (Figure 2B). Immunohistochemical stains were positive for Vimentin, SMA, CD31 (Figure 2C), ERG, and FLI1, while CD34 (Figure 2D), chromogranin, synaptophysin, desmin, CKAE1/3, CK7, TTF-1, ER/PR, and human herpes virus-8 (HHV-8) were all negative. Morphological features were those of vascular neoplasm-SCH with no overt features of malignancy. A pathological diagnosis of SCH was thus made. On follow-up six months after the surgery, the patient was asymptomatic.

## Discussion

In 1986, Enzinger et al. described a new variant of vascular tumor which they proposed as “spindle cell hemangioendothelioma” with limited malignant potential [1]. A detailed review of 78 cases by Perkins et al. evaluated its pathogenesis and long-term behavior. It affected all ages (range 8-78 years, median age 32 years, mean age 34 years) and both genders equally [2]. The tumor presented as a superficial mass in the upper (32 cases) or lower (30 cases) extremities. The lesions were circumscribed red-brown masses occasionally containing phleboliths and consisted of cavernous blood spaces alternating with cellular areas having collapsed vascular spaces separated by spindled fibroblastic cells. The characteristic appearance comprised endothelial cells (epithelioid with cytoplasmic vacuolization) and spindled fibroblastic cells (lacking significant atypia and with low mitotic activity). The majority of the tumors (58%) were intravascular growing into the lumen as multifocal lesions or contiguous lesions. Despite conservative excisions in most patients, the prognosis was excellent with no metastasis or death attributed to SCH, although a high local recurrence rate (58%) was described. The authors concluded that SCH was a primary benign vascular neoplasm or malformation similar to angiomatosis in which alterations in blood flow might explain some of the secondary features. Areas of diminished blood flow result in vascular collapse with the formation of the “cellular” zones, and areas of vascular engorgement with stasis that promote thrombosis and organization.

Per previous reports, SCH most commonly arises in the sub-cutis in distal extremities or in mucous membranes, specifically the oral mucosa. Case reports describe SCH in muscles, retroperitoneum, mediastinum, and spinal cord [3]. Per our literature search, only one case of SCH in the lung has been reported in the English literature [1]. In that instance, SCH presented as multiple round nodules with smooth borders and uniform density in both lungs, and the diagnosis was made by video-assisted thoracoscopic biopsy of the largest and most accessible lesion.

In our case, SCH presented as a single enlarging pulmonary nodule on a background of multiple smaller stable nodules and ground-glass opacities. In contrast to the previously reported case of pulmonary SCH, which described findings at a single time point, we demonstrate the slow evolution of an SCH lesion over many years. The largest nodule in our patient slowly increased in size and changed its morphology from smooth to irregular density and speculated margins; these radiologic features suggest a typical malignant lesion. Histology of our patient’s lesion showed no evidence of malignancy; rather, features were characteristic of SCH. This is in accordance with the literature describing difficulty in preoperative diagnosis of SCH, with diagnosis relying primarily on postoperative pathological analysis and immunohistochemistry [4].

The major differential diagnosis of pulmonary nodule includes - malignancy, infectious/non-infectious granuloma, tuberculosis, and hamartoma [5]. The patient’s prior history of breast cancer and her smoking history increased her risk for metastasis or primary lung cancer [6]. To make a definitive diagnosis, a right middle lung lobectomy was done. Based on pathological analysis and immunohistochemistry studies, both metastatic and primary carcinomas were excluded. Negative synaptophysin and chromogranin did not support spindle cell carcinoid. Histologic findings and positive staining with Vimentin, SMA, CD31, ERG, and FLI1 showed characteristic features of SCH [7]. Although Kaposi sarcoma or Kaposi-like hemangioendothelioma were also in the differential, morphological features and negative HHV-8 staining ruled these out as well.

As noted previously, only one previous report of pulmonary SCH has been described in the literature. Consequently, there is no consensus on the management and prognosis of pulmonary SCHs. Thus, the management philosophy could be borrowed by extrapolating the management of SCH elsewhere. SCH is primarily treated by local resection, and although a high recurrence rate is noted, no long-term metastases or mortality has been attributed to SCH themselves. Thus, local resection of pulmonary nodule when considered suspicious should be done as deemed necessary, and upon diagnosis of SCH, no further radiation or chemotherapy should be needed.

## Conclusions

SCHs are benign vascular neoplasms that present as red-purple nodules under the skin or mucus membrane of young adults and have a similar incidence in both sexes. Only one reported instance of SCH in the lungs presenting as bilateral, round lesions with smooth margins were found in the literature. To the best of our knowledge, this is only the second case of pulmonary SCH, and the first to show the progressive growth and morphologic change over time. In both cases, a diagnosis of SCH was made only after undergoing surgical resection of the suspicious pulmonary nodule. As pulmonary SCH seems to present as pulmonary nodules, a decision for radiologic monitoring and surgical intervention should follow the widely agreed upon Fleischner society guidelines. Once diagnosed, surgical resection should suffice and there should be no need for additional surgery, chemotherapy, or radiation therapy. This case report broadens the differential diagnosis of enlarging and irregularly contoured pulmonary nodules.
